# Reactive stroma component COL6A1 is upregulated in castration-resistant prostate cancer and promotes tumor growth

**DOI:** 10.18632/oncotarget.3697

**Published:** 2015-03-30

**Authors:** Yi-Ping Zhu, Fang-Ning Wan, Yi-Jun Shen, Hong-Kai Wang, Gui-Ming Zhang, Ding-Wei Ye

**Affiliations:** ^1^ Department of Urology, Fudan University Shanghai Cancer Center, Fudan University, Shanghai, China; ^2^ Department of Oncology, Shanghai Medical College, Fudan University, Shanghai, China

**Keywords:** castration-resistant prostate cancer, COL6A1, reactive stroma, tumorigenesis

## Abstract

Castration-resistant prostate cancer (CRPC) remains the most critical challenge in the clinical management of prostate cancer (PCa). Reactive stromal changes in PCa are likely involved in the emergence of CRPC. In the present study, we identified a novel oncogene termed COL6A1 which was upregulated in the reactive stroma of CRPC. We established an androgen-independent LNCaP (LNCaP-AI) cell line in steroid-reduced (SR) medium within 2 months. We examined COL6A1 expression with western blot during the LNCaP-AI induction, and studied the function of COL6A1 *in vitro* and *in vivo*. Immunohistochemical staining of COL6A1 was performed in ten pairs of androgen-sensitive PCa and CRPC samples. We demonstrated that COL6A1 expression was markedly increased in LNCaP-AI cells and CRPC tissues compared with LNCaP cells and paired androgen-sensitive PCa specimens. *In vitro*, COL6A1 knockdown resulted in G1-S cell cycle arrest and descended vitality. Overexpression of COL6A1 was associated with accelerated S phase entry and elevated vitality in prostate cancer cells. COL6A1 also promoted tumorigenesis of LNCaP cells *in vivo*. Taken together, these data suggest an important role of COL6A1 in the molecular etiology of castration-resistant prostate cancer, and support the potential use of COL6A1 in CRPC therapy.

## INTRODUCTION

Androgen-deprivation therapy (ADT) is typically employed as the first-line treatment for locally advanced or metastatic prostate cancer (PCa). For most patients, the initial responses are usually positive within approximately 2 years. After that, the tumors frequently recur and become castration-resistant, with a correspondingly poor prognosis.

Considerable recent efforts toward elucidating the molecular mechanisms of castration-resistant prostate cancer (CRPC) suggest that stromal-epithelial interactions play a key role in response to castration therapy [1]. The tumor-associated stroma within the cancer is called reactive stroma. Trichrome stain makes it easy to distinguish normal stroma from reactive stroma. Smooth-muscle-rich prostatic normal stroma stains red; fiber-rich reactive stroma presents as a predominance of blue staining, because the fiber architecture of reactive stroma becomes disorganized and is much smaller than that of normal stroma [2]. Previous studies reported that reactive stroma was associated with advanced tumor stage in PCa [3] and could be used as a predictor of reduced recurrence-free survival [4, 5]. Further studies suggested that the reactive stroma surrounding prostate tumor lesions performed critical roles, including supporting tumor cell proliferation and inducing tumorigenesis and metastasis [2].

The altered extracellular matrix components of reactive stromal in prostate tumors are collagen-rich compared with normal stroma [2]. However, the component and function of collagens in reactive stroma remain unclear. We assume that collagen components in reactive stroma may be altered during the transformation to castration resistance. By analyzing sequencing results from published data [6] and validating these results with our experiments, we identified COL6A1, a novel gene which encoded collagen six subunit alpha 1 and was upregulated in CRPC. We demonstrated for the first time that COL6A1 could promote tumor proliferation *in vitro* and *in vivo*, suggesting its potential useful application to CRPC therapy.

## RESULTS

### The establishment of LNCaP-AI cells

**Figure 1:**
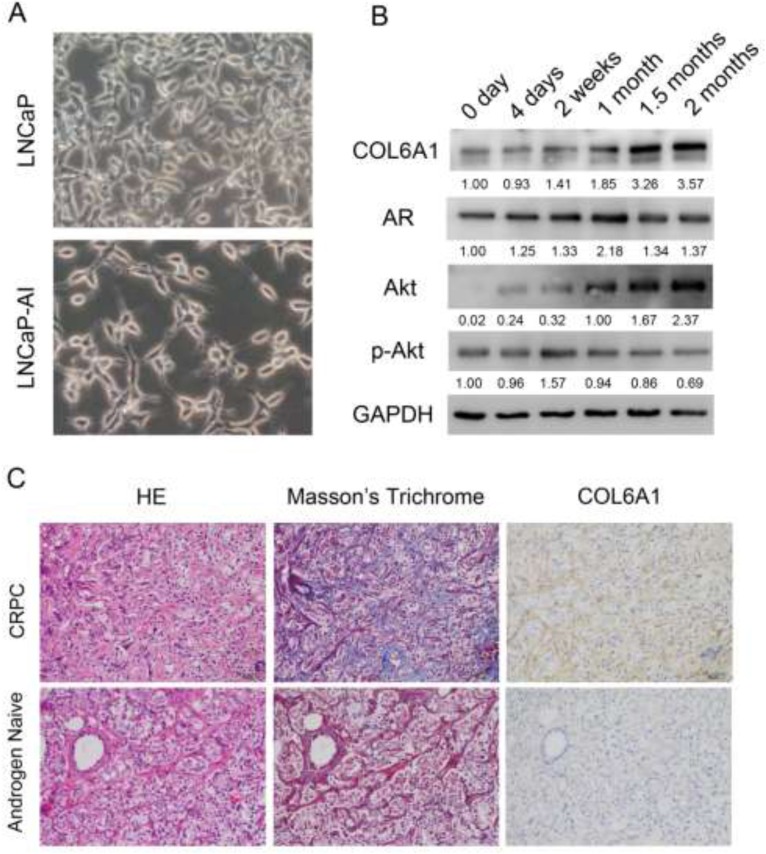
**A**, the 400X photographs of LNCaP and LNCaP-AI. LNCaP-AI cells exhibited a neuronal morphology with compactly rounded cell bodies, having extended and fine-branched processes. **B**, COL6A1 is upregulated in induced castration-resistant LNCaP cells. The proteins were harvested at 0 day, 4 days, 2 weeks, 1 month, 1.5 months, and 2 months after induction of SR conditions. The expression of COL6A1, AR, Akt, and p-Akt were examined by western blot. COL6A1 and AR and Akt were upregulated during castration. However, p-Akt level was stable or even decreased during castration. **C**, COL6A1 is overexpressed in patients with CRPC. In Masson's trichrome staining, collagen stains blue and cytoplasm stains red. Thus, collagen-rich tumor stroma (blue) can be differentiated from normal stroma (red, because preexisting normal host stroma is rich in smooth muscle bundles). The picture of H&E, Masson's trichrome stain, and IHC of COL6A1 of paired androgen-naïve and patients with CRPC were photographed in 200X. The reactive stroma and COL6A1 expression were significantly upregulated in patients with CRPC.

After being cultured in SR medium for approximately 2 months, the LNCaP-AI cells grow stably and much faster than when they are first placed into the medium. LNCaP cells have an epithelial morphology, tapering into unbranched processes that are generally shorter than the cell body. LNCaP-AI cells exhibit a neuronal morphology with compactly rounded cell bodies, having extended and fine-branched processes (Figure 1A).

### COL6A1 was upregulated in LNCaP-AI cells and CRPC specimens

To examine the alterations of COL6A1 expression in LNCaP-AI cells, we conducted western blots of parental androgen-dependent LNCaP cells, androgen-independent LNCaP-AI cells, and the series of SR-treated LNCaP cells at different time points. COL6A1 expression was upregulated stepwise under SR conditions (Figure 1B). We then analyzed the IHC scores of COL6A1 in androgen-dependent prostate cancer (ADPC) and CRPC specimens (Figure 1C). We included a total of 20 specimens from 10 individuals in the analysis. COL6A1 was upregulated in CRPC specimens (mean COL6A1 score 3.4 CRPC vs. 2.3 ADPC, Table 1, *p* < 0.05). The tumor stroma was also enriched in many CPRC specimens. Interestingly, the reactive stroma grade (RSG) of CRPC specimens was higher than that of ADPC specimens (mean score 1.8 vs. 0.2, *P* < 0.01).

**Table 1:** Clinical characteristics of 10 Pca patients

	At Diagnosis						TURP at CRPC					
Case No.	Age	PSA	GS	Stage	COL6A1 score	RSG	Age	PSA	GS	Stage at TURP	COL6A1 score	RSG
1	70	4.8	5+4	cTxNxM1b	2	0	77	11.48	5+5	cT4NxM1b	3	1
2	58	35	3+4	cT3bN1M1b	2	0	63	85.61	4+4	cT4N1M1b	4	0
3	67	46.11	4+4	cT4N1M1c	2	1	77	361.11	4+4	cT4N1M1c	4	2
4	74	50	4+4	cT4N1M1b	2	0	81	37.1	5+4	cT4N1M1b	4	2
5	73	35	5+5	cT3bNxM1b	3	1	77	7.56	5+5	cT4NxM1b	2	2
6	52	364.6	3+5	cT4N1M1b	2	0	54	111	4+5	cT4N1M1b	3	1
7	74	19.08	5+5	cTxNxM1b	3	0	76	34.3	5+5	cT4NxM1b	4	3
8	61	305	5+5	cT4N1M1b	2	0	62	150	5+5	cT4N1M1b	3	2
9	70	69	4+4	cT3bN1M1c	3	0	78	57.11	5+4	cT4N1M1c	4	2
10	63	140	4+4	cT4N1M1b	2	0	66	10.59	5+5	cT4NxM1b	3	3

GS Gleason score, TURP transurethral resection of prostate. RSG, reactive stroma grade: grade 0,<5%; grade 1,5% to 15%, grade 2, 15% to 50%, grade 3, >50%[4].

### COL6A1 silencing inhibited prostate cancer cell proliferation

**Figure 2:**
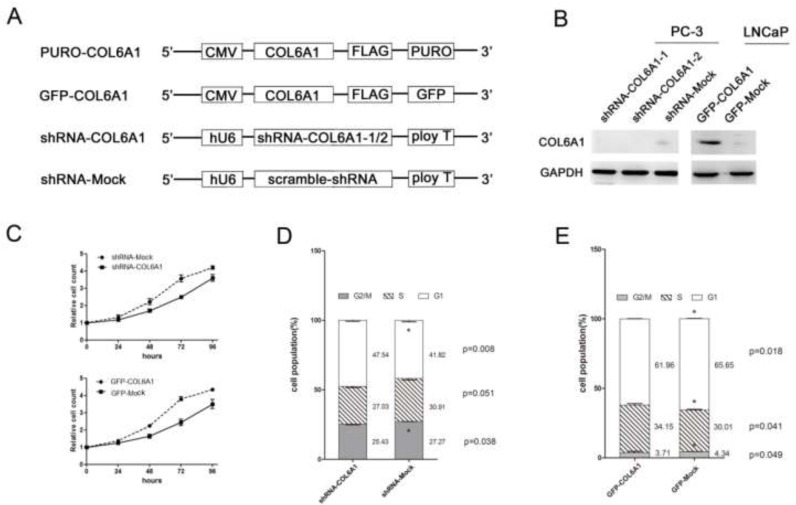
COL6A1 could decrease cell proliferation in prostate cancer cells **A**, All vectors used in this study are shown above. COL6A1 overexpression constructs were inserted after CMV promoter of pCDH-CMV-MCS-EF1-Puro (CD510B-1) or pCDH-CMV-MCS-EF1-copGFP (CD511B-1). pLKO.1 TRC cloning vector (Addgene Plasmid 10878) was employed to generate shRNA constructs against COL6A1. **B**, The COL6A1 expressions of all stable cell lines was examined by western blot. COL6A1 was successfully knocked down in PC-3 cells and was successfully overexpressed in LNCaP cells. **C**, CCK-8 assay was used to detect the proliferation capability of stable transfected cell lines. **D**, **E**, The cell cycle alteration of COL6A1 knock-down or overexpression in stable cell lines was detected by flow cytometry.

To study the role of COL6A1 in cell growth, we analyzed the PCa cell lines that were treated with COL6A1 ShRNA. COL6A1 ShRNA 1-3 were designed according to the RNAi Consortium (TRC)-provided clones using vector plKO.1 (Figure 2A). Western blot analysis showed that sh-COL6A1-1 and shCOL6A1-2 exhibited the most effective knockdown effect (Figure 2B). Results suggested that silencing COL6A1 resulted in an obvious accumulation of PC3 cells in G1/S phase (FACS) in PC-3 cells and in growth inhibition (CCK-8 assay; Figure 2C and 2D). COL6A1 did not demonstrate a significant effect on apoptosis (data not shown). Consistent with COL6A1 inactivation, PC-3 cells lost their rapid proliferation ability. These results suggest that COL6A1 knockdown could inhibit proliferation in PCa cells.

### COL6A1 upregulation promoted prostate cancer cell proliferation

To further clarify the role of COL6A1 in prostate cancer, we constructed overexpression vectors containing an open reading frame (ORF) of COL6A1 and used lentivirus to introduce the vectors into LNCaP cells which barely expressed COL6A1. Western blot analysis showed that the amount of COL6A1 in these cells was considerably higher than it was in controls (Figure 2B). LNCaP cells transfected with GFP-COL6A1 vectors exhibited fewer cells in G1/G0 phase, more cells in S phase (FACS assay), and increased survival (CCK-8), compared with the negative controls (Figure 2C and 2E). These results suggest that artificially induced exogenous COL6A1 could promote the proliferation of prostate cancer cells. COL6A1 may play an important role in the progression of prostate cancer.

### Overexpression of COL6A1 could promote tumorigenesis in nude mice

**Figure 3:**
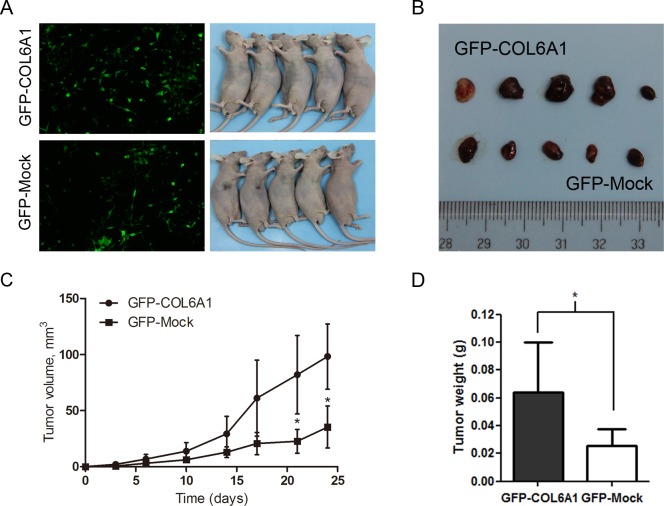
Overexpression of COL6A1 promoted tumor growth in nude mice **A**, We stably infected LNCaP with the GFP-COL6A1 overexpression and control particles to upregulate COL6A1 level. The stable infected cells were bilaterally injected into 5 nude mice. **B**, **D**, The mice were humanely killed on day 27, and the tumors were photographed and weighed (* represents *P* < 0.05). **C**, COL6A1 overexpression mediated increased tumor growth of LNCaP cells in nude mice. Tumor volume (mean SD [error bars]) was measured with a Vernier caliper on days 3, 6, 10, 14, 17, 21, and 24.

To determine whether COL6A1 is correlated with tumorigenesis *in vivo*, we stably infected LNCaP with the lentiviral COL6A1 and mock particles to overexpress COL6A1. The results showed that COL6A1 is effectively overexpressed (Figure 3A). After subcutaneously injecting nude mice with the stably infected cells, we measured tumor volume with a Vernier caliper twice a week (mean SD [error bars]). The tumor volume of the COL6A1-transfected tumor was significantly larger than that of the Mock-transfected tumor on day 21 (33.38 vs. 89.05mm^3^, *p* < 0.05) and day 24 (42.02 vs.127.32mm^3^, *p* < 0.05; Figure 3C). The tumor masses from mice treated with COL6A1- overexpressed particles were greater than those of the mock-treated mice when the tumors were harvested and weighed on day 27 (Figure 3B and 3D). Together, these results demonstrated that COL6A1 might act as an oncogene, which could promote prostate cancer proliferation.

### COL6A1 silencing inhibited prostate cancer cell proliferation via the JAK2-STATs pathway

**Figure 4:**
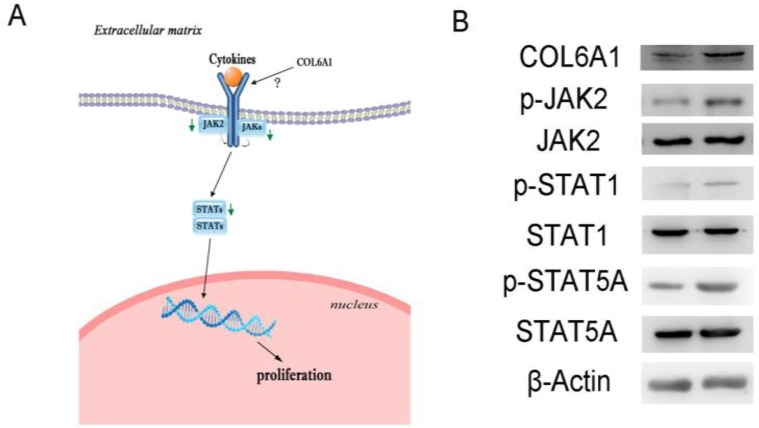
Signal pathway alterations were examined by protein microarray **A**, Green arrows indicate blockaded pathways. The JAK2-STATs pathway was blockaded and may be responsible for decreased downstream gene expression, inhibiting cell proliferation. **B**, Microarray results of shRNA-COL6A1 PC-3 cells (left) were validation by western blots, compared to shRNA-mock PC-3 cells (right).

In the Cancer Signaling Phospho Antibody Microarray PCS248 analysis of COL6A1 silencing, the JAK2-STATs pathway was the most significantly changed (Figure 4A). COL6A1 silencing inhibited JAK2 (Phospho-Tyr221, fold change 0.80) and downstream STATs. STAT1 (Phospho-Tyr727, 0.70) and STAT5A (Phospho-Ser694, fold change 0.62) were the most significantly dephosphorylated. These findings were validated by western blot. p-JAK2, p-STAT1, and p-STAT5A were inhibited with COL6A1 silencing in PC-3 cells (Figure 4B).

## DISCUSSION

Although encouraging new drugs like abiraterone and enzalutamide have been recently developed, the prognosis of patients with CRPC remains poor. The underlying mechanism of ADPC progression into CRPC is a hot topic, although it remains poorly understood. In the present study, we identified COL6A1, a novel gene which was upregulated in LNCaP-AI cells and CRPC specimens. Silencing COL6A1 inhibited PC-3 proliferation and led to G1 cell cycle arrest. In addition, COL6A1 overexpression promoted PCa cell proliferation through cell cycle activation. In a mouse model of prostate cancer, COL6A1 influenced the growth of tumors from nude mice subcutaneously injected with LNCaP cells. Finally, we determined and validated that knockdown of COL6A1 could inhibit JAK-STATs pathway activities.

COL6A1 is one of the three major subunits of collagen VI, which is an important extracellular matrix (ECM) protein that interacts with other molecules in ECM and cell membranes, providing structural support for cells [7, 8]. This subunit is involved in multiple signaling pathways that regulate apoptosis [9], proliferation, angiogenesis, fibrosis, and inflammation [10, 11]. Recent studies indicate that COL6A1 is differentially expressed in tumors and adjacent normal tissue [12], and is associated with tumor progression [13]. Other researchers have used transcriptome profiling methods to demonstrate that COL6A1 is reactivated in CRPC [6]. Our research supports these previous findings and further reveals the function of COL6A1 in PCa. However, the mechanism that underlies its involvement in CRPC remains unclear.

Our IHC results showed that COL6A1 mainly existed in PCa tumor stroma, also known as reactive stroma [14]. In prostate cancer, reactive stroma consists of a mixture of fibroblasts, myofibroblasts, nerves, endothelial cells, immune cells, and altered ECM, and is distinct from the normal mesenchymal tissues of the prostate. Previous studies showed that marked reactive stroma formation was associated with poor outcome in clinically localized prostate cancer [14, 15]. Men with tumors that have reactive stroma grade 3 (RSG3), the most profound histologic alterations of reactive stroma, have reduced biochemical recurrence-free survival, and/or increased prostate cancer-specific death [4, 16, 17]. We found that the reactive stroma components increased during the transformation from ADPC to CRPC. COL6A1 that is synthesized and secreted by prostate cancer cells may play a role in or act like a marker for the transformation from ADPC to CRPC.

Our results showed that COL6A1 played an important role in tumor proliferation and might influence the transformation from ADPC to CRPC. We discovered that downregulation of COL6A1 could inhibit the cell proliferation, mainly by the JAK-STATs pathway, and we validated these results. Previous studies of the well-established JAK-STATs signaling pathway showed that JAK-STATs blockade led to inhibition of specific gene transcription and decreased cell proliferation [18-20]. Further study showed that JAK-STAT pathway components could influence the prognosis of patients with PCa [21]. STATs act as transcription factors and could produce sophisticated biological effects. Further mechanisms of action must be explored.

There are several limitations to this study. This was a single-center pilot study, thus possible selection bias and less statistic power were unavoidable. Therefore, further validation of our work at other centers and including patients of other ethnicities is needed. We also did not illustrate how COL6A1 acts on the JAK-STATs pathway, directly or indirectly. IHC analysis cannot specifically differentiate between the contributions of different sources of extracellular secretion (i.e., tumor cells, stroma cells, normal prostate gland cells). New technology, like the RNA scope, could probably provide insights into the specific location of COL6A1 expression and improve our understanding of the extracellular environment. However, this study did reveal a novel gene related to reactive stroma that reactivated in CRPC and for the first time illustrated the function of COL6A1 in PCa. Our research may prompt further studies of how the reactive stroma interacts with prostate cancer and how this could trigger CRPC.

## MATERIALS AND METHODS

### Patients and clinical samples

This study was approved by the Institutional Research Review Board of Fudan University Shanghai Cancer Center (FUSCC), and carefully followed both ethical and procedural rules for clinical studies, including documented informed consent obtained from all subjects in this study. Patient data and clinical samples were collected from June 2006 to December 2012. Following androgen deprivation therapy at FUSCC, patients diagnosed with metastatic PCa (*n*=79) were subjected to transurethral resection of the prostate (TURP) for dysuria Ten patients received TURP for dysuria when diagnosed with CRPC. Ten paired formalin-fixed paraffin-embedded (FFPE) samples of androgen-sensitive PCa and CRPC tumors were obtained from the FUSCC tissue bank. In addition, we retrospectively reviewed the medical records. Table 1 displayed the clinical, pathological characteristics of the patients with PCa.

### The index tumor and immunohistochemistry (IHC) analysis

A single experienced FUSCC pathologist identified the index tumor, defined as the largest and/or highest Gleason tumor and most likely to be clinically significant, on all hematoxylin and eosin (HE) slides. The pathologist was blinded to the clinical data. We selected slices representative of the highest and most common Gleason rate and homologous FFPE specimens for use in the analysis. IHC analyses to evaluate COL6A1 expression in FFPE specimens were carried out as previously described [22]. The IHC result of COL6A1 was reviewed with respect to staining intensity and extent. A staining index (range, 0–9) was obtained as a product of staining intensity (range 0–3) and percentage of positive cells (≤1%, 0; 1%–25%, 1; 25%–50%, 2; >50%, 3). Cutoff points were defined using four grades: grade 1, 0–2; grade 2, 3–4; grade 3, 5–7; grade 4, 8–9. Grade 1 was considered negative [23]. To accurately differentiate between tumor stroma and normal pre-existing host stroma, we took an adjacent slice to find tumor-reactive stroma and stained with Masson's trichrome before evaluating the COL6A1 expression level with IHC, as per previous reports [4, 24, 25].

### Cell lines

The PCa cell lines PC-3 (CLR-1435) and LNCaP (CRL-1740) were obtained from American Type Culture Collection (ATCC) and cultured as follows: LNCaP in RPMI 1640 (L0495, Biowest, Logan, UT, USA), PC-3 in F-12K (21127-022, Life Technologies, Carlsbad, CA, USA) plus 10% fetal bovine serum (FBS; S181P, Biowest) in a humidified incubator with a 5% CO_2_ atmosphere at 37ºC.

### Establishment of LNCaP-AI subline

We established stable LNCaP-AI cells by culturing androgen-sensitive LNCaP cells in androgen-reduced conditions resembling clinical androgen ablation therapy, as previously described [26]. LNCaP cells were initially seeded in regular RPMI 1640 complete medium for 3 days, and then were continuously maintained in an SR medium (Phenol Red negative RPMI 1640 medium with 10% certified charcoal/dextran-treated FBS) with a testosterone concentration of less than 0.1 nM from Hyclone (Logan, UT, USA) and 100 mg/L bicalutamide (B9061, Sigma Chemical Co., MO, USA). Under these conditions, the cell growth diminished and the morphology gradually changed into a neuroendocrine-like phenotype. The LNCaP-AI cells grew faster after 2 months in SR conditions. We harvested these cells at 4 days, 2 weeks, 1 month, 1.5 months, and 2 months after induction of SR conditions. We used LNCaP cells that were maintained in the regular medium as controls.

### Plasmid construction and *in vitro* study

We used pLKO.1 TRC cloning vector (Addgene Plasmid 10878) to generate shRNA constructs against COL6A1 [27]. The sequences of shRNA targeting COL6A1 are listed in Table S1. We inserted the CDS sequence of COL6A1 followed by 3′ FLAG tag sequence into pCDH-CMV-MCS-EF1-Puro (CD510B-1) or pCDH-CMV-MCS-EF1-copGFP (CD511B-1) vectors. Lentiviral particles were produced in HEK293T cells by co-transfection of pLKO.1-shCOL6A1 constructs with packaging vectors psPAX2 and pMD2.G. Stable cell lines expressing shRNAs were obtained by lentiviral infection followed with puromycin selection [28-31]. Proteins were harvested after selection of stable infected cells and SR-treated cells (different time points as described above). Western blots were analyzed using anti-COL6A1 antibody (17023-1-AP, Proteintech, Chicago, IL, USA). The relative levels of proteins were quantified using densitometry with the Gel-Pro Analyzer (Media Cybernetics, Rockville, MD, USA). The target bands over the GAPDH band were densitometrically quantified and indicated under each band.

In CCK-8 assays, cells were counted and seeded 1,000 cells per well in 96-well plates. Seeded plates were incubated for 24, 48, 72, and 96 h in a humidified incubator with a 5% CO_2_ atmosphere at 37ºC. We then added 10 ul CCK-8 (5mg/ml; Dojindo, Japan) to each well and incubated the plates for 2 h at 37°C. The number of cells was then assessed by measurement of absorbance at 450 nm using an ELx800Absorbance Microplate Reader (BioTech, Winooski, VT) [32]. In flow cytometry assays, selected stable infected cells were harvested and fixed with −20°C pre-cooling 70% ethanol overnight at 4°C. Cells were then washed 3 times with cold PBS, and stained with propidium iodide (PI) 50 mg/ml at room temperature for 15 min. PI-stained samples were run on an FC500-MPL Beckman Coulter and the cell cycle data were analyzed using MultiCycle AV DNA Analysis software [32]. Apoptosis assays were conducted using a Dead Cell Apoptosis Kit with Alexa (V13241, Invitrogen, CA, USA).

### *In vivo* study

We stably infected LNCaP cells with the lentiviral COL6A1-GFP particles to increase the expression of COL6A1. We collected GFP-Mock Control LNCaP and GFP-COL6A1-LNCaP cells and subcutaneously injected 5×10^6^ cells/point into the right and left flanks of nude mice (5 total), respectively. To monitor growth, we used a Vernier caliper to measure the tumors on days 3, 6, 10, 14, 17, 21, and 24, and calculated the volumes as W^2^×L×0.5 (where Wand L represents the largest and next largest tumor diameters in cm), then plotted the values. Mice were humanely sacrificed on day 27, and the tumors were weighed and photographed.

### Phosphoprotein profiling by the cancer signaling phospho antibody microarray and validation

To further reveal the mechanisms of how COL6A1 may influence prostate cancer proliferation, we performed the Cancer Signaling Phospho Antibody microarray PCS248, which was designed and manufactured by Full Moon Biosystems, Inc. (Sunnyvale, CA) and contains 248 antibodies. Each of the antibodies has two replicates that are printed on coated glass microscope slides, along with multiple positive and negative controls. The antibody array experiment was performed by Wayen Biotechnology (Shanghai, China), according to their established protocol. The fluorescence signal of each antibody was obtained from the fluorescence intensity of this antibody spot. A ratio computation was used to measure the extent of protein phosphorylation. The phosphorylation ratio was calculated as follows: phosphorylation ratio = phospho value / unphospho value. Validations were performed by western blot using the following antibodies: anti-JAK2 antibody (ab68269, Abcam), anti-JAK2 (phospho Y221) antibody (ab196009), anti-STAT1 antibody (ab2415), anti-STAT1 (phospho S727) antibody (ab109461), anti-STAT5a antibody (ab32043), anti-STAT5 (phosphoY694) antibody, anti-COL6A1 antibody (17023-1-AP, Proteintech, Chicago, IL, USA), and anti-beta-actin antibody (20536-1-AP, Proteintech, Chicago, IL, USA).

### Statistical analysis

All statistical analysis was completed using SPSS version 17.0 (SPSS, Inc.). Continuous variables were analyzed using independent t tests. Categorical variables were analyzed with Pearson's Χ^2^ tests. *P*<0.05 was considered statistically significant. In accordance with previous reports, a 12% phosphoprotein ratio downregulation or fold change >2 were marked as significant [33, 34].

## SUPPLEMENTARY MATERIAL TABLE


