# Supplementary material for: Reactive stroma component COL6A1 is upregulated in castration-resistant prostate cancer and promotes tumor growth
Source: Oncotarget. 2015 Mar 30;6(16):14488–96. doi: 10.18632/oncotarget.3697 (PMC4546481; doi:10.18632/oncotarget.3697)
Supplement: Supplementary file 1 [file oncotarget-06-14488-s001.pdf]

Reactive stroma component COL6A1 is upregulated in castration-resistant prostate cancer and promotes tumor growth

Supplementary Material

Table S1,The sequences of shRNAs targeting COL6A1.

Primer	Direction	Sequence（5’-3’）
shCOL6A1-1	Forward	CCGGGTGGGCATCAAAGACGTGTTTCTCGAGAAACACGTCTTTGATGCCCACTTTTTG
	Reverse	AATTCAAAAAGTGGGCATCAAAGACGTGTTTCTCGAGAAACACGTCTTTGATGCCCAC
shCOL6A1-2	Forward	CCGGCAAAGTCAAGTCCTTCACCAACTCGAGTTGGTGAAGGACTTGACTTTGTTTTTG
	Reverse	AATTCAAAAACAAAGTCAAGTCCTTCACCAACTCGAGTTGGTGAAGGACTTGACTTTG
shCOL6A1C3	Forward	CCGGGCTGTGTCTTACTAGAAACAACTCGAGTTGTTTCTAGTAAGACACAGCTTTTTG
	Reverse	AATTCAAAAAGCTGTGTCTTACTAGAAACAACTCGAGTTGTTTCTAGTAAGACACAGC
